# Rethinking the Theory of Change for Health in All Policies: Comment on "Health Promotion at Local Level in Norway: The Use of Public Health Coordinators and Health Overviews to Promote Fair Distribution Among Social Groups"

**DOI:** 10.15171/ijhpm.2018.96

**Published:** 2018-10-01

**Authors:** Ditte Heering Holt

**Affiliations:** National Institute of Public Health, University of Southern Denmark, Copenhagen, Denmark.

**Keywords:** HiAP, Health Equity, Municipalities, SDH, Theory of Change

## Abstract

This commentary discusses the interesting and surprising findings by Hagen and colleagues, focusing on the role of the public health coordinator as a Health in All Policies (HiAP) tool. The original article finds a negative association between the employment of public health coordinators in Norwegian municipalities and consideration of a fair distribution of social and economic resources between social groups in local policymaking and planning. The commentary contemplates whether this surprising negative association should be interpreted as a failure of implementation, as suggested by the authors, or whether it might be the theory of change that has failed. On this basis, it is suggested that the very notion of HiAP could be flawed by the assumption that health should function as an overarching aim across government sectors. Potentially, the social determinants of health (SDH) might be more efficiently addressed by means of sectoral action by the corresponding sectors, emphasizing equity rather than health.

The study by Hagen et al^1^ investigates the relationships between changes in municipal use of Health in All Policies (HiAP) tools and the extent to which fair distribution of social and economic resources between social groups is taken into account in local policy-making. The study finds that 70% of the Norwegian municipalities considered fair distribution among social groups in their local health promotion initiatives, while only 38% of the municipalities considered fair distribution in their general policy-making. Surprisingly, the study finds that employment of public health coordinators was negatively associated with fair distribution. The study thus raises questions regarding whether institutional changes, such as employing a public health coordinator, are appropriate HiAP tools. This commentary discusses the role of public health coordinators and how we might interpret the surprising results by Hagen and colleagues. By introducing a distinction between implementation failure and failure of the theory of change from theory-based evaluation,^2^ it is suggested that it may not be sufficient to consider this surprising result as a case of implementation going wrong. We should also consider whether the theory of change has failed. That is, maybe the very notion of HiAP is flawed by the assumption that health should function as an overarching aim?

## The Public Health Coordinators

In the article, Hagen and colleagues^1^ suggest that some explanation (to the negative association between public health coordinators and fair distribution between social groups) may be found in the high number of health coordinators who are not employed full time. This entails that the public health coordinators do not have the time required to adequately facilitate collaboration and coordination and engage in advocacy, planning, and policy-making. Another explanation suggested by Hagen and colleagues is that many public health coordinators are not placed in the office of the chief executive officer (CEO) but often in the department of the medical officer, and thus not in a position of power to influence local policy-making. These explanations suggest that the negative association between public health coordinators and a fair distribution between social groups should be understood as a case of implementation failure, in the sense that had the function of public health coordinator been properly implemented (employed in a full-time position in the central CEO office) it would have been successful.

However, while it is often believed that bringing policy teams or specialized groups to ‘the center’ of government can provide the uptake and integration of health concerns across government, a recent study^3^ among Danish municipalities found that the organization of public health teams (employing public health coordinators) in central units, did not strengthen their coordination and oversight role or imbue them with the authority to change policy and practice across the diverse portfolios of local government as was assumed. Instead, the public health officers’ problems with gaining traction among non-health departments were mostly reproduced. While different ways of organizing public health held different advantages and disadvantages, most municipalities had not managed to solve the overall problem of establishing more widespread and profound intersectoral commitment and decision-making for health.^3^ Thus, placing public health coordinators in the central CEO office may not necessarily provide the institutional power needed to affect the agenda of local policy-making. This is also supported by Scheele et al^4^ who found that no Scandinavian municipality has been fully successful in terms of organizing and establishing governance for health equity.

Moreover, though it is very reasonable to assume that a part-time position is not enough to ensure the necessary time to facilitate collaboration and ensure an intersectoral commitment for health across sectors, in the Danish study we found that bigger units, with everything between two and ten public health coordinators, did not manage to create more profound intersectoral support and commitment for public health aims across government. Thus, ensuring enough time for the public health coordinator to fulfill its role, might not be enough to ensure the desired results. Internationally, the systematic review by Carey et al^5^ also problematizes the ability to create change in government by embedding public health coordinators in the government bureaucracy if they are not backed by strong political support at multiple levels and a supportive architecture providing accountability and incentive mechanisms, among others.

Another potential implementation failure worth considering is whether the public health coordinators possess the competencies and skills required to function in the role as facilitators of collaboration and coordination they have been assigned. The competencies to undertake the role as boundary spanner (that is the facilitating role of establishing collaboration and coordination across sectoral and departmental boundaries to ensure joined-up working) are rather different from traditional public health competences and requires a different set of skills. In the study by Hagen et al,^1^ it is neither clear which educational backgrounds the public health coordinators have, nor for which specific skills they have been selected to hold the position. However, others have highlighted how the public health community does not have a good understanding of the policy process and may lack necessary skills.^5^ For instance, de Leeuw et al^6^ argue that many health promoters tend to see policy change through the lens of intervention research, which generally applies a linear understanding of change that is quite different from the ‘messy’ world of policy. Carey and Crammond^7^ have found that health promoters who work to create policy change need to be able to break down the ‘problem’ (of the social determinants of health, SDH) into parts, which correlate with the structures of government. That is, the problems need to fit within specific departmental boundaries as well as the accountability and incentive structures of government.^7^ Williams^8,9^ refers to this role of the boundary spanner as a communicator and interpreter. This involves the ability to translate public health aims into relevant boundary issues together with an appreciation of otherness, which encompasses empathizing with and respecting the different cultures, motivations, gazes, and practices of a wide range of professionals from different sectors. The toolbox of the public health coordinators may thus need to be expanded to include the skills required for successful joining up.

Altogether, part-time employment in the department of the medical officer (rather than the CEO office) of public health coordinators, who might lack training in boundary spanning and policy processes, may help explain the lack of success of this HiAP tool. However, if we were to conceptualize this finding as an implementation failure, in the sense that the public health coordinators simply do not have the resources (time and mandate) or the skills to create change, one would expect no significant differences between the municipalities who have employed a public health coordinator and the municipalities that do not meet this recommendation. Yet, it does not seem to sufficiently explain the strong negative association between public health coordinators and fair distribution between social groups identified by Hagen et al.^1^ Thus, in order to better understand this surprising result, we may need to consider a more critical perspective when discussing the appropriateness of the public health coordinators as a HiAP tool. This entails questioning the theory of change of HiAP itself.

## The Theory of Change of HiAP

HiAP is often defined as “an approach to public policies across sectors that systematically takes into account the health implications of decisions, seeks synergies, and avoids harmful health impacts in order to improve population health and health equity.”^10^ It entails the notion that by introducing governance structures and institutional arrangements (such as public health coordinators), governments will be able to achieve a whole-of-government approach to ensure better population health and health equity.^5^ In the words of Hagen and colleagues, “HiAP aims to foster an explicit health focus through the ‘whole-of-government’”^1^ (p. 2). As such, HiAP aspire to make health accepted as an overarching aim of government, and the public health coordinator’s key task is to facilitate collaboration and coordination to ensure this.

However, this theory of change has several weaknesses, the main one being the assumption that non-health sectors would generally accept health as an overarching aim. This opposes the sectoral reality of governments, where the different sectors enable government to break down the complexity of governing.^7^ As Degeling^11^ has argued: “while ‘the better coordination and cooperation’ envisaged in these calls [for intersectoralism] has great moral appeal, this does not mean that the special interests involved will be more disposed to become what they are not: that is, to divest themselves of their bias and take on a more universal and hence for them, more formless perspective”^11^ (p. 295). In this perspective, the call for intersectoralism in public health is naïve, because sectors purposefully represent institutionalized mobilizations of different interests and values. Following Degeling, we should consider such bias (ie, different interests and values) not simply as an error, which can be erased by intersectoral structures, but as normal and integral to structuring human interaction and thus inescapable. As phrased by Exworthy and Hunter^12^: “it is presumptuous to think that health (inequalities) might trump other equally compelling deserving causes.” In other words, health is not a key priority of non-health sectors,^3,7^ and a theory of change based on the assumption that institutional arrangements can provide the uptake and integration of health concerns across government is most likely to fail.

When considering how this may affect the public health coordinators’ ability to effect change, a study among municipalities implementing the Norwegian Public Health Act is interesting. Synnevåg et al^13^ reported how several informants in Norwegian municipalities found it inappropriate for public health to take ownership of sectoral operations, as practitioners already carried out the kind of work referred to with the public health terminology in their daily operations as teachers, social workers, and urban planners, “thus questioning the need to call it public health work” (p. 70). Consequently, Synnevåg and colleagues cautioned the risk of health imperialism if all local policy-making is labelled to be a matter of health.

Following this line of argument, the negative association between public health coordinators and the consideration of a fair distribution between social groups, found by Hagen et al,^1^ may (partly) be explained by public health coordinators pursuing the HiAP strategy of introducing health as an overarching aim (and thus creating resistance among non-health sectors), rather than promoting the sectoral contributions of these sectors that would ensure a fair distribution of SDH.

This seems to be the case elsewhere. For instance, in a former study^14^ we found that efforts to integrate health across local government sectors translated into a limited approach where interventions tended to favor smaller-scale behavioral interventions aimed at creating healthy settings, while neglecting the distribution of broader SDH. While this was done to ensure legitimacy for intersectoral policy-making for health and thus avoid resistance from non-health sectors, it resulted in a lifestyle drift and a risk of corrupting the SDH.^14^ The numbers of Hagen et al,^1^ where public health coordinators are negatively associated with fair distribution among social groups, and only 38% of the municipalities prioritize fair distribution in their general policy-making, could reflect a similar dynamic. Even the very ambitious HiAP strategy introduced in South Australia appears to have failed to address health equity.^15^ Consequently, it is worth considering whether (some) HiAP tools, such as public health coordinators, may risk being counterproductive for the goal of ensuring better health equity.

## Time to Ditch the H Word?

It is generally agreed that the SDH comprise the circumstances in which people are born, grow, live, work, and age, and the unequal distribution of these determinants is considered to be a driver of health inequity.^16,17^ This includes factors such as income and poverty, education, employment, housing, and the built environment among others.

As such, if we want to ensure action on the SDH, it might be time to reconsider HiAP’s theory of change in terms of the assumption that health should be an overarching aim across government. Rather, the main contribution of non-health sectors would be to ensure equity concerns be included in sectoral policies and practice in education, employment, and social services, among others. As such, the SDH might be more efficiently addressed by means of sectoral action in various non-health sectors, emphasizing equity rather than health. This may find support in the studies by Scheele et al^4^ and Synnevåg et al^13^ who found that an emphasis on “social sustainability” or “living conditions” may function better to establish support across government sectors. Thus, if the aim is a fair distribution of social and economic resources between social groups in general policy-making, it might be time to stop framing the conversation to be focused on health. Instead, we should acknowledge that health equity is a political and ideological matter about equity,^11,18,19^ rather than simply a matter of government organization waiting to be fixed using HiAP tools, such as the employment of public health coordinators.

As for HiAP’s theory of change, I hope to see more studies testing and critically assessing to what extent (if at all) HiAP and its various institutional arrangements are efficient at changing government policies in favor of (health) equity. This remains an imperative question for further research.

## Ethical issues

Not applicable.

## Competing interests

Author declares that she has no competing interests.

## Author’s contribution

DHH is the single author of the paper.
